# A guide to megastudies

**DOI:** 10.1093/pnasnexus/pgac214

**Published:** 2022-10-07

**Authors:** Angela L Duckworth, Katherine L Milkman

**Affiliations:** Department of Operations, Information and Decisions, The Wharton School, University of Pennsylvania, Philadelphia, PA 19104, USA; Department of Psychology, School of Arts and Sciences, University of Pennsylvania, Philadelphia, PA 19104-6018, USA; Department of Operations, Information and Decisions, The Wharton School, University of Pennsylvania, Philadelphia, PA 19104, USA

## Abstract

How can behavioral insights best be leveraged to solve pressing policy challenges? Because research studies are typically designed to test the validity of a particular idea, surprisingly little is known about the relative efficacy of different approaches to changing behavior in any given policy context. We discuss megastudies as a research approach that can surmount this and other obstacles to developing optimal behaviorally informed policy interventions. We define a megastudy as “a massive field experiment in which many different treatments are tested synchronously in one large sample using a common, objectively measured outcome.” We summarize this apples-to-apples approach to research and lay out recommendations, limitations, and promising future directions for scholars who might want to conduct or evaluate megastudies.

Significance Statement From voter turnout to vaccine adoption, public policy challenges of all kinds benefit from effective interventions for changing human behavior. The aim of a conventional intervention study is to test a single approach—not to compare the efficacy of different interventions. To address this lack of comparability, we propose a new paradigm: megastudies are massive field experiments in which many different treatments are tested synchronously in a large sample using a common objective outcome. We summarize this apples-to-apples approach to research and lay out recommendations, limitations, and promising future directions for scholars who might want to conduct or evaluate megastudies.

## Introduction

How can we best leverage behavioral insights to solve pressing policy challenges? For instance, how can behavioral science effectively help governments and organizations improve individuals’ decisions about whether or not to get vaccinated, exercise, stay in school, and save money? Despite an exponential increase in individual studies on behaviorally informed policy tools over the last decade (1), it is often unclear which behavioral insights are most relevant to a specific policy challenge. Why? Typically, individual research studies are designed to establish the validity of a single idea, not to assess its efficacy relative to other theoretically informed approaches in a particular policy context. We propose that the megastudy approach surmounts this and many other obstacles to developing optimal behaviorally informed policy interventions (2).

## The megastudy paradigm

**Fig. 1.**
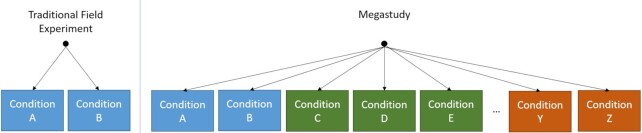
Left panel: traditional field experiments randomly assign participants to multiple conditions (e.g. Conditions A and B), testing a limited number of related hypotheses. Right panel: megastudies randomly assign participants to a larger set of treatments often clustered by sub-study (different colors indicate different sub-studies), each testing potentially unrelated hypotheses (e.g. Conditions A, B, C, D, etc.).

We define a megastudy as “a massive field experiment in which many different treatments are tested synchronously in one large sample using a common, objectively measured outcome” [(2), p. 479]. Megastudies typically take the form of independent research teams developing sets of treatment(s) and control conditions (“sub-studies”), with participants randomly assigned across all of them. To qualify as a megastudy, a field experiment should have a variety of different conditions; the more numerous and diverse the conditions, the more appropriate it is to classify the experiment as a megastudy. See Fig. 1.

The megastudy paradigm builds on ideas that have been used to improve other scientific disciplines. The common task framework, for example, radically advanced machine learning in the past two decades (3). In this framework, scientists compete to solve a common problem (e.g. image classification) using the same data set, optimizing the same performance outcome and operating under the same constraints. Scientific tournaments have a similar flavor, although they do not typically involve random assignment of participants to condition (4). Likewise, meta-analyses compare results across studies executed by different scientists, but they use different samples in different settings and often compare different outcome measures (5).

Megastudies afford several distinctive advantages.

First and foremost, megastudies enable true apples-to-apples comparisons of different behavioral interventions in terms of both efficacy and cost effectiveness. As a result, they can determine which tool has the highest expected value for addressing a pressing policy problem (6). In addition, megastudies accelerate the speed of scientific progress by testing many different hypotheses simultaneously rather than serially. And because they include a wide range of interventions in a very large sample of well-characterized participants, megastudies are ideally suited for advanced computational analyses of what works best for whom over a wide range of levels of different independent variables and under what conditions [(7,8); Goldstein (9].

Because the considerable time and expense necessitated by field research are borne by a central organizer, megastudies also make possible efficiencies of scale that can dramatically lower the marginal cost of field studies for individual research teams. Relatedly, lowering the barriers to entry to ecologically valid field research can increase the number of scientists who can conduct policy-relevant behavioral science research. Megastudies also address the “silo problem” (10,11) by bringing together researchers from diverse methodological and theoretical backgrounds to address a single policy-relevant problem [see (12)].

Finally, because it is a diversified portfolio of sub-studies, a megastudy hedges the risk of any individual sub-study failing to yield publishable results. This can incentivize researchers to take greater risks with the ideas they develop and test. Likewise, megastudies make it straightforward to publish null results, reducing the file drawer problem (13), and clarifying the frequency at which null results occur so accurate conclusions about overall effectiveness can be drawn (Beshears and Kosowsky (14); Mertens et al. (1); Maier et al. (15); Bakdash et al. (16); Szaszi et al. (17)).

## An illustrative megastudy: improving vaccine adoption

One early megastudy was motivated by the urgent need to encourage vaccine adoption during a global pandemic. In partnership with Walmart Pharmacy, researchers tested the efficacy of 22 different text messages, compared with each other and with a business-as-usual control (i.e. no-message) condition, which encouraged patients to get their flu shot in fall 2020 (18).

In March 2020, the lead megastudy investigators sent a request for proposals to roughly 100 behavioral scientists, inviting them to submit sub-study designs. More than 30 designs were submitted, nine of which were selected for inclusion in the megastudy based on feasibility (assessed by the megastudy leaders in collaboration with Walmart), redundancy (similar ideas were merged), and forecasted potential for impact (as assessed by the megastudy leaders). In total, 28 teams of scientists—psychologists, economists, and computer scientists—designed 22 different conditions in the 9 final sub-study designs.

Nearly 700,000 Walmart Pharmacy patients were randomly assigned across conditions, with roughly equal probabilities of receiving messages determined by their treatment condition, or the business-as-usual condition. Vaccine adherence was measured in the preregistered 3 month period from September 2020 (when patients received texts) to December 2020.

All treatment conditions significantly outperformed the business-as-usual control condition, demonstrating the value of text messages nudging vaccine uptake. In addition, in analyses exploring the underlying attributes of more successful messages, we found that treatments, including multiple messages significantly outperformed those including a single message, confirming that repeated reminders add value.

The top-performing treatment communicated to patients that a vaccine was “waiting for you.” And this was the message we recommended for widespread use to encourage vaccination. Attribute analyses indicated that reminders containing “ownership” language generally outperformed other messages, and two additional studies confirmed the value of using similar messages in text reminders to encourage vaccination against both the flu and COVID-19 (19,20). Follow-up experiments exploring the mechanism responsible for this effect demonstrated that it conveyed a sense of exclusivity, which contributed to its benefits (21).

## Megastudy best practices

### Preparation for study launch

To an extent, the best practices for running a megastudy are the same as those for any field experiment (22). Ideally, a megastudy targets a policy-relevant and objectively measurable outcome variable. Given the possibility of selective attrition, this outcome should be measurable for every participant, thereby enabling intent-to-treat analyses. For instance, gym attendance or vaccine receipt at a given retailer are objectively measurable outcomes that an organizational partner can provide for all participants regardless of attrition. In contrast, an outcome measure like step count, while objectively measurable, requires participants to synchronize a pedometer, and therefore, participant motivation may affect measurement.

An ideal organizational partner for a megastudy is highly motivated to change the target outcome and appreciates the power of the scientific method, ensuring incentive alignment and mutual understanding. A legal agreement should be negotiated with the partner, including how data will be shared, a division of roles and responsibilities, and explicit permission for the publication of results.

After identifying an ideal organizational partner, the parameters for the megastudy should be agreed upon in collaboration with this partner. These parameters include the communications (e.g. text messages, emails, and mailings) and incentives that researchers can design, as well as the cadence and time period for their deployment. In addition, a megastudy control condition to which all conditions can be compared should also be designed. Throughout, it is imperative to maintain a high-trust relationship with a single primary contact in the partnering organization, reducing the likelihood of miscommunication and methodological errors in implementation.

A megastudy request for proposals should be developed that includes the aforementioned information as well as the anticipated sample size for each treatment arm, determined based on power calculations that account for multiple comparisons across the megastudy, and the expected detectable impact of the interventions tested. To assure that the nuances of the megastudy parameters are communicated successfully with study designers, it can be especially useful to supplement this written information with as many informational sessions and one-on-one conversations as necessary.

The universe of potential researchers can be entirely open or, to limit the number of submissions that will need to be processed, be restricted to members of a specific academic (or nonacademic) community. Megastudy leaders and staff should screen these initial submissions, identifying ideas similar enough to be merged, and selecting a subset to advance to the organizational partner to evaluate their feasibility (in terms of both legality and execution). Finally, organizers can make final selections either by reviewing and selecting submissions or using a lottery.

Ideally, following best practices in open science, analysis plans for all sub-studies in the megastudy should be preregistered, and an analysis plan for the megastudy itself should also be preregistered prior to launch [Banks et al. (23); (24)]. Likewise, within the constraints agreed upon with organizational partners, megastudy data should be shared publicly for secondary analyses.

### Analyzing data

The more conditions a megastudy includes, the more feasible it is to run analyses identifying the common attributes of effective conditions. Preregistered attribute analyses should specify whether attributes are objectively coded (e.g. word count) or subjectively rated (e.g. the message content was surprising) by a separate sample of participants, ideally one demographically similar to the megastudy sample. [See (18) for an example megastudy attribute analysis that led to a replication and extension by (19), in an independent sample.]

Because megastudies include many treatment arms, correcting for multiple hypothesis testing is necessary in analyses. There are, of course, many different approaches for doing so. In Milkman et al. (18), we used the Benjamini–Hochberg procedure to adjust *P*-values to control the false discovery rate (i.e. the expected fraction of true nulls among the set of results declared to be significantly different from zero) (25). Unlike some alternative approaches that adjust for the false discovery rate, this procedure accounts for the fact that interventions are compared to a common control condition and, hence, results of these comparisons are positively correlated.

Further, while the most effective treatment should be recommended to policy makers, some caution is warranted. Because of the winner’s curse (26,27), the magnitude of its treatment effect is likely to be overestimated. Therefore, the top-performing condition’s estimated treatment effect should be adjusted downward by applying a correction such as the James–Stein shrinkage procedure (28).

## Limitations

The scale of megastudies brings drawbacks as well as benefits. One is their considerable fixed cost. While there are gains in efficiency from scale, a megastudy may not be feasible if resources (e.g. budget, personnel) are limited. Our hope, of course, is that funding agencies and foundations prioritize megastudies in the future. Likewise, while megastudies diversify the portfolio of ideas tested, they also increase the risk of an implementation failure affecting not just one experiment but many sub-studies (and research teams). The scale of megastudies also limits their replicability and requires megastudy leaders who are motivated to not only test their own ideas but also accept ideas of other scientists. Megastudies also tend to require enormous participant samples. Human behavior is hard to change, and given realistic estimates of treatment effects (29), adequate statistical power requires tens if not hundreds of thousands of participants.

Not all policy solutions are amenable to examination by field experiment or megastudy, and, of course, many of the most efficacious solutions may not be. For instance, addressing climate change will require changes in corporate incentives, carbon taxes, and international treaties [see (30)]. In general, it is more feasible in a megastudy to randomly assign individuals to alternative communications, social interactions, and incentives than to alternative laws and policies.

By necessity, conducting a megastudy requires strict enforcement of study design parameters (e.g. the number of text messages that will be sent). While the rigidity of these parameters enables apples-to-apples comparisons, it also limits innovation (e.g. creative ideas that do not adhere to the megastudy constraints).

Another limitation of megastudies is that they can give rise to inclusion issues. Which researchers will have the resources to launch megastudies and who will be invited to contribute ideas? If the institutions funding megastudies and the groups of researchers invited to contribute sub-study designs are exclusive, this may exacerbate existing gaps between the haves and have-nots in academia ((31); Nielsen & Andersen (32)).

## Future directions

The megastudies that have been conducted to date have primarily examined whether variations in online activities, microincentives, text messages, and emails delivered to individuals can change behavior over periods of up to 1 or 2 months (20, 33). Future megastudies could test more social and experiential interventions (e.g. creating or assigning groups of individuals to meet and carry out prescribed activities) and aim to treat participants for longer time horizons [Rogers & Allcott, (34)]. Laboratory-based megastudies could also be used to probe questions of interest in controlled environments, and some such studies have already been done [e.g. Della Vigna and Pope, (35); (36)].

To date, megastudies have solicited intervention ideas “bottom up” from researchers without attempting to systematically explore the theoretical intervention space. Future megastudies might attempt to map out differences between treatments varied in a theoretically motivated way (36). For example, such megastudies might systematically vary incentive size, contact frequency, message length, or other dimensions of theoretical interest.

Existing megastudies have yet to yield exciting advances in researchers’ understanding of what treatments work best for whom. With innovations in machine learning and increasingly large study samples (37), future megastudies should further explore this frontier (7,8).

Relatedly, in future megastudies, adaptive random assignment could be used to direct additional participants to more promising treatment arms based on early data collection, thus better powering those treatments that have the greatest potential (38). In megastudies to date, merely executing balanced random assignment across many treatment arms has proved challenging for many partners, but with advances in technology and as familiarity with the megastudy methodology grows, more sophisticated random assignment processes should become possible.

Finally, by definition, a megastudy compares interventions that are randomly assigned and synchronously executed. But there is an urgent need to enable piloting and iterative prototyping (39; Berman & Bulte 40; Azevedo et al. 41), ideally in a subsample from the target population or a demographically similar parallel sample.

## Conclusion

Megastudies are a promising new tool for identifying the behavioral insights most likely to help address pressing policy problems. To be clear, megastudies should not replace standard field experiments, which are far more appropriate when researchers seek to evaluate a single hypothesis. However, when policy makers need to choose one or two solutions for an urgent behavioral problem, testing many ideas simultaneously in a megastudy can ensure they deploy the most cost-effective tools available. In other words, megastudies are one way for behavioral science to be more solution-focused (12).

## Data Availability

All data are included in the manuscript and/or supporting information.
